# The psychometric profile of chiropractic patients in Norway and England: using and comparing the generic versions of the STarT Back 5-item screening tool and the Bournemouth Questionnaire

**DOI:** 10.1186/2045-709X-21-41

**Published:** 2013-11-23

**Authors:** Pernille Irgens, Lise R Lothe, Ole Christian Kvammen, Jonathan Field, David Newell

**Affiliations:** 1 Private practice Grimstad Kiropraktorklinikk, Grimstad, Norway; 2 Private practice Fokus Helse og Trening, Sandefjord, Norway; 3 Network of chiropractic research clinics in Norway, KIP, Oslo, Norway; 4 Private practice Back2 Health, Petersfield, UK; 5 Anglo-European College of Chiropractic, Bournemouth, UK

**Keywords:** Start back tool, Bournemouth questionnaire, Musculoskeletal pain, Screening tools

## Abstract

**Background:**

Musculoskeletal pain and low back pain (LBP) in particular is one of the more costly health challenges to society. The STarT Back Tool (SBT) has been developed in the UK with a view to identifying subgroups of LBP patients in order to guide more cost effective care decisions. The Bournemouth Questionnaire (BQ) is a validated multidimensional patient reported outcome measure (PROM) that is widely used in routine clinical practice settings. This study sets out to describe and compare SBT and BQ scores within and between populations of patients presenting for chiropractic care in Norway and Great Britain.

**Methods:**

Patient demographics, BQ and the 5-item generic condition SBT data were collected from patients presenting with musculoskeletal pain to 18 Norwegian and 12 English chiropractors. Analysis of correlation between groups was achieved using a 1-way Chi2 approximation (p < 0.05).

**Results:**

Eleven percent of Norwegian LBP patients (n = 214) and 24% of English LBP patients (n = 186) were “distressed by their condition” (SBT > 4). By comparison, Norwegian chiropractic patients are: somewhat younger, have lower BQ scores, are less distressed by the condition and score significantly lower on items relating to catastrophisation and depression than English patients. There was an apparent association between total BQ and SBT scores (correlation 0.59, p < .0001) and patients who scored higher than 45 (IQR 39–58) on BQ were more likely to respond “distressed by condition” (>4) on SBT. Furthermore, patients in “distressed by condition” SBT category who had marked the “low mood” question on SBT also had a high score on the “depression” question of BQ (>6 (IQR 4–8), correlation 0.54, p < .0001).

**Conclusion:**

The BQ and SBT appear to identify the same subgroups in some, but not all of the measured items. It appears that unknown factors result in variations between patients seeking chiropractic care for comparable complaints in primary care in England vs Norway. Comparison of populations from Norway and UK demonstrate that extrapolating and pooling of data in relation to different populations should be done with caution, in regard to these stratification tools.

## Introduction

Musculoskeletal pain, and low back pain (LBP) in particular, is widely recognized as being one of the more costly health challenges to our society [1]. Despite significant research efforts to provide more insight into the causes of musculoskeletal pain, many questions remain unanswered. It is widely recognized that a large portion of LBP patients possess no clear pathoanatomic etiology for their symptoms, with around 80% falling into the nonspecific category [2]. Waddell [3] espoused a change from the traditional pathoanatomical approach to the management of LBP to a biopsychosocial model. Some authors have proposed a more prognostic approach in contrast to a diagnostic [4]. A combination of biopsychosocial and pathoanatomical models of understanding seems to be appropriate [1].

Various screening tools have been developed to identify subgroups and factors of comorbidity in regard to musculoskeletal pain patients [5-7] However, not all of these approaches are easy to apply which can be a barrier to use in clinical practice. There are few psychosocial questionnaires that have been validated for use on all musculoskeletal pain patients presenting to primary care and that are designed to collect baseline data before examination and diagnosis by a health care provider and before the therapeutic alliance has been formed. This paper explores the use of two short comprehensive psychosocial measurement tools that have been validated for specific conditions and where the developers have made a generic non-validated version available for use in all musculoskeletal pain conditions; the STarT Back Tool (SBT) [5] and the Bournemouth Questionnaire (BQ) [8].

The SBT is a validated stratification tool for back pain designed to identify subgroups of patients in order to guide the practitioner to early prevention in primary care. This questionnaire is easy and efficient to use for the clinicians, and specially designed to help in clinical decision-making [5]. The SBT aims to easily stratify the patient into three subgroups that define their probability of poor prognosis, or chronicity. These categories are defined as “Low risk”, “Medium risk”, and “High risk”. The original SBT was a diagnosis-specific screening tool, for use on LBP patients presenting to General Practitioners in England. Several adaptations have later been made, as well as several validated translations. The strength of SBT is that it is designed for clinicians, and that the three allocation groups indicate specific treatment strategies for the patients. A recent paper indicates the superiority in outcome for LBP patients, allocated to different treatments by the use of SBT [5]. SBT developers recommend the high risk patients to specific intervention that combines physical and psychological modalities [9]. The SBT is available in 16 languages through the Keele University website [10]. We used the Norwegian version of the tool that was available through the official site at the time of data-collection. This version was translated but to our knowledge not formally validated. This translation has been tested for face validity but this has not yet been published in a peer-reviewed journal.

The Bournemouth Questionnaire (BQ), is a validated questionnaire, developed for use in routine practice settings in back [8] and neck [11] pain patients, and is widely used in chiropractic clinical settings and research. It focuses on pain and disability from a biopsychosocial model in that musculoskeletal pain is a complex and multicomponent entity. The questionnaire is a multidimensional outcome instrument designed to measure pain, disability, anxiety, depression, fear-avoidance and locus of control [8]. The BQ pre-treatment questionnaire has been found to predict low back pain and risk of sick leave one year after initial treatment by chiropractors [12]. It is however designed to measure change and as such it can be used as a tool predicting outcome based on early change [13]. BQ has been translated to Danish, Dutch, German and French for use in LBP [8,14-16] and neck pain [11,17-20] conditions. The developers’ state on their website that “The validated BQ 7 scale instrument can be downloaded for the following conditions: back pain; neck pain and general musculoskeletal pain”. There is also a link to the English language versions of these instruments [21]. It is unclear whether the general musculoskeletal pain questionnaire available has been validated.

This study investigated the ability of the SBT to categorize patients and assess whether there was an association between SBT and BQ scores for LBP patients presenting to chiropractors in UK and Norway. The study also aimed to identify the demographics and basic psychological profile of patients presenting to chiropractic practice in Norway.

## Methods

This is a cross-sectional study of baseline data collected in primary care settings in two countries. Data were collected from a network of Norwegian chiropractic clinics and from data routinely collected electronically by a group of chiropractic clinics in southern England (UK).

### Translation of the English version of the Bournemouth Questionnaire

A generic version of the BQ for use in all musculoskeletal pain conditions has been developed in English but has to our knowledge not undergone translation or validation. The BQ back pain questionnaire has been translated to Norwegian as part of a large multi-center predictor study [6,12,22] but the validation process has not been published. For the predictor study the BQ was first translated into Norwegian and then back translated to English by two different professional translators. The BQ developer compared the original and back-translated English versions for content and wording. The Norwegian version was then tested for patient usability on six subsequent treatment visits in 12 low back pain (LBP) patients. The change in BQ score was compared to the clinician’s record and found to reflect clinical change (unpublished appendix in the predictor study).

Our study utilized the Norwegian translated LBP BQ from the predictor study [12] as a template for the generic condition version we wanted to use. A group of clinicians who are members of the Norwegian clinical research network (KIP) compared the Norwegian LBP BQ to the English version of the generic BQ downloaded from the developer’s website [10]. All clinicians were experienced chiropractors and knowledgeable in British, Australian or American English. The translated versions were discussed, and adjusted to obtain consensus and close equivalence with the original version. Specifically, “painful complaint” was translated to “*smerteproblem”,* “complaint” to “*plage*”, and “anxious” to “*urolig*”. Back-translation was performed by two bilingual individuals with knowledge of Norwegian and English at a professional academic level, and with English as a native language. The original and the back-translated English versions of the generic BQ were later compared by three clinicians. If discrepancies were found, the Norwegian version was adjusted to optimize conceptual overlap. The translated generic BQ version was tested on Norwegian speaking patients presenting to a chiropractor, and no particular problems in answering the questions were reported.

### STarT back tool

We used the 5-item STarT Back generic condition tool which is the 9-item psychosocial subscale modified to screen and identify distress in other conditions [5]. The questions addresses; fear (1 item from the Tampa Scale of Kinesiophobia), anxiety (1 item from the Hospital Anxiety and Depression Scale), pessimistic patient expectations (1 item from the Pain Catastrophizing Scale), low mood, (1 item from the Hospital Anxiety and Depression Scale) and how much the patient is bothered by their pain [7]. All items use a response format of ‘agree’ or ‘disagree’, with exception to the bothersomeness item, which uses a Likert scale. Scores range from 0 to 5 and patients scoring 4 or 5 are being classified as high psychosocial risk. We used the five psychometric questions from the 9-item Norwegian SBT version available from the Keele University website April 2012 [10].

### Procedure Norwegian population

Objectives and practical procedures were explained to the participating chiropractors at a meeting for a network of chiropractic clinics volunteering in data collection for research where valid data collection and use of questionnaires in clinical decision-making was the topic. All patients who presented to a chiropractor with a painful complaint and were above the age of 18 and who were able to read and write Norwegian were invited to participate. Patients were excluded if they had attended a chiropractic consultation or received treatment less than 3 months prior to the initial visit. The SBT and BQ questionnaires were completed in the waiting room prior to first visit. After the consultation the chiropractor chose a diagnosis among a list of 26 commonly used diagnoses used in Norwegian chiropractic clinics based on the ICPC (International Classification of Primary Care) codes reported to the National Insurance Scheme of the Norwegian Labour and Welfare Service. The de-identified questionnaires were then transferred into an online survey tool (Web survey creator, Dipolar Pty Limited, Sydney, Australia) either by a secretary or the chiropractor. The paper forms were sent to the coordinator to check accuracy of data entry. The 26 diagnoses were further compiled into four areas of complaint, back, neck, extremity and other for convenience of analysis and comparison with other studies.

### Procedure UK

The STB and BQ are routinely collected in the participating UK chiropractic clinics through a free online system (Care Response, London) accessed via any internet browser to help health care providers gather and report clinical outcome and patient satisfaction information and which has been previously described [23]. The data is stored in a database with secure encryption, which gives clinicians access only to information that relates to only their clinic. Data were collected with explicit consent to be used anonymously for research purposes.

The initial assessment completed by patients included a brief background medical history, and the 9-item SBT and BQ. A summary was provided for their practitioner. This included calculation of the SBT ranking of the patient’s risk of having disabling pain in three months. Outcome forms are automatically sent to patients by email 14, 30 and 90 days after starting care but for this study we only used the baseline data pertaining to the five psychosocial items. Data from LBP patients with the same inclusion criteria as the Norwegian participants with initial consultation during the month of May 2012 were extracted for analysis.

### Ethics

The study was conducted in accordance with the Declaration of Helsinki, registered with the Norwegian Data Protection Authority (registration no 47214) and was deemed exempt from ethics committee approval. No variation was made to patient care as a consequence of this study and all participants consented to anonymous data being used for research purposes.

### Data analysis

Demographics (age, sex, duration and area of pain) are presented as a simple distribution. The SBT was scored according to the methods specified by the instrument developers [10] and classified as “distressed by condition” if SBT score >4. BQ was scored on a 0–10 point scale for each question with a maximum total score of 70. Associations between the SBT and BQ were calculated using Spearman’s rank correlations in JMP 10, SAS institute Inc., Cary, NC, USA and in IBM SPSS 17 Statistics, IBM Corporation, Somers, NY, USA. The magnitude of the correlation coefficient was evaluated as small if 0.1-0.3, moderate if 0.3-0.5, and high if greater than 0.5 [24]. The Chi-squared test was used for categorical data and results given in medians with interquartile range (IQR) unless otherwise specified. We used an alpha level of .05 for all statistical tests.

## Results

Data were collected electronically through Care Response from all low back pain patients visiting twelve chiropractors in six practices in Southern England. From Norway 26 chiropractors were invited to the study. Eighteen participated and of those, six logged the assigned 20 paper forms collected from consecutive pain patients into the relevant online data base, three logged more than 20 and nine logged less than 20 (range 2–60 datasets). The average time to log patient data was 58 seconds. All data were collected from a total of 510 patients in the month of May, 2012.

Twenty-six different musculoskeletal diagnoses were used and these were reduced to four areas of complaint; back, neck, extremity and other musculoskeletal complaint (Table 1). Neck pain included cervicogenic headache and jaw pain. The back pain group included thoracic back pain (n = 2) and low back pain with radiating extremity pain (n = 96). Other musculoskeletal complaints that were not area specific (i.e. fibromyalgia) were grouped together in an “Other” category. Duration of back pain complaint was similar between counties with 37% chronic (>12 weeks) and 45% acute (<3 weeks) chi-square test X^2^ (N = 398) = 0.57, p = 0.8. There were more females seeking care than men although not significantly different between countries for back pain chi-square test X^2^ (N = 398) = 0.72, p = 0.4 (Table 1).

**Table 1:** Patient characteristics at baseline

		**Norway**				**England**
		**Other**	**Extremity**	**Neck**	**Back**	**Back**
Gender N F (%)		6 (54.5)	20 (62.5)	39 (57.3%)	113 (53.1)	107 (57.3)
Age N(%)						
	18-24	2	3 (9.4)	7 (10.3%)	20 (9.3)	8 (4.3)
	25-34	3	5 (15.6)	19 (27.9)	42 (19.6)	21 (11.4)
	35-44	2	6 (18.8)	20 (29.4)	66 (30.8)	37 (20.0)
	45-54	2	9 (28.2)	14 (20.6)	45 (21.0)	51 (27.7)
	55-64	3	4 (12.5)	2 (2.9)	25 (11.7)	34 (18.3)
	>65	1	5 (15.6)	6 (8.8)	16 (7.5)	34 (18.3)
Duration (weeks) N (%)						
	<1	3	1 (3.2)	13 (19.1)	52 (24.3)	54 (29.2)
	1-2	2	6 (18.8)	9 (13.2)	34 (15.9)	23 (12.4)
	2-3	1	0.0	4 (5.9)	21 (9.8)	9 (4.9)
	3-12	2	8 (25.0)	15 (39.7)	33 (15.4)	30 (16.2)
	>12	3	17 (53.1)	27 (39.7)	72 (34.6)	69 (37.3)
Duration category N (%)						
	Acute	6	7 (21.9)	26 (38.2)	107 (50.0)	86 (46.5)
	Sub acute	2	8 (25.0)	15 (22.1)	33 (15.4)	30 (16.1)
	Chronic	3	17 (53.1)	27 (39.7)	74 (34.6)	69 (37.3)
Total N (%)		11 (3.4)	32 (9.8)	68 (20.9)	214 (65.8)	185 (100)

Table 1 show the demographic profile of patients presenting to the participating chiropractic clinics in Norway and LBP UK patients.

**Figure 1:**
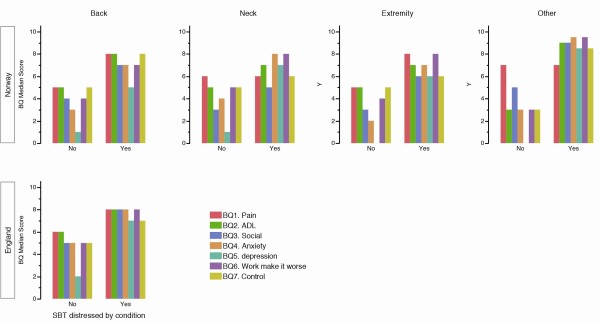
**The median score for the different BQ items in participants that according to SBT are “distressed by condition” (SBT ≥ 4) and in participants that are not (SBT < 4).** Note the low score for BQ depression in participants with a low SBT total score for all areas of complaint.

The Norwegian patient population was younger than the English chi-square test X^2^(N = 398 ) = 26.6, p < 0.0001* (Table 1). The English population had a higher total score on the SBT and a majority agreed to the catastrophizing and depression statements bringing the distressed by condition (SBT > 4) to 24% of the population compared to 11% of the Norwegian low back pain population chi-square test X^2^(N = 398 ) = 12.9, p = 0.0003. BQ total score were also higher in the English back pain population 37(24–49) versus 31 (20–40) in Norway chi-square test X^2^(N = 398 ) = 3.8, p < 0.0001. In the Norwegian population sixteen percent of the extremity and neck pain patients reported being distressed by condition, and 18% of the others (Table 2). In the Norwegian population the BQ total score was higher in both the back 31(20–40) and other 31(17–31) pain area groups than in the extremity 29(14–40) and neck 22(20–40) pain populations. The patients classified by SBT as being distressed by condition had a higher score on all BQ items than patients classified by SBT as not being distressed by condition (Table 3 and Figure 1).

**Table 2:** STarT back score

	**Norway**				**England**	p^* by country^
SBT items (Mean score %)	Other	Extremity	Neck	Back	Back	
Not safe active (Y)	18.2	9.4	8.8	14	12.6	0.69
Worrying thoughts (Y)	63.3	68.8	48.5	41.6	32	0.05
Never improve (Y)	27.3	34.4	22.5	22	58.7	0.0001
Not enjoy things (Y)	18.18	18.8	29.4	18.7	58.7	0.0001
Bothersome >3 (Y)	72.7	68.8	61.8	57.9	55.7	0.65
Distressed by condition (Y)	18.2	15.6	16.2	10.8	24.2	0.004

* = Chi2.

Table 2 Mean score for the different STarT Back items for different areas of complaint. Note the high score for pessimistic patient expectations and low mood items in the UK LBP population compared to the Norwegian.

**Table 3:** Median (IQR) BQ sub-score for patients distressed by condition SBT 5-item psychosocial score >4

				**BQ items**							
		**(STB > 4)**	**n**	**Pain**	**ADL physical**	**ADL social**	**Anxiety**	**Depression**	**Work**	**Control**	**Total BQ**
**Norway**	Neck	Yes	57	6(6–8)†	7(6–8)	4(2–5)	8(3–8)	7(4–9)	5(4–10)	6(4–9)†	45(37–58)
		No	11	6(4–7)†	5(3–7)	2(0–3)	4(3–6)	1(0–4)	5(2–7)	5(3–7)†	29(21–38)
	Extremity	Yes	5	8(7–9)	7(5–9)†	3(2–6)†	7(5–8)	6(4–7)	5(3–8)	6(5–10)†	45(34–61)
		No	27	5(4–7)	5(2–7)†	3(0–7)†	2(0–5)	0(0–2)	4(1–6)	5(3–6)†	26(13–38)
	Other	Yes	2	7(6–8)†	9(8–10)	8(8–9)†	10(9–10)	9(8–9)	10(9–10)	9(9–10)†	61(58–64)
		No	7	7(4–7)	3(3–6)	5(2-5	3(2–6)	0(0–3)	3(4–4)	3(2–7)†	26(17–34)
	Back	Yes	23	8(7–9)	8(6–9)	5(5–8)	7(5–8)	5(2–8)	7(6–9)	8(6–8)	43(39–53)
		No	193	5(4–7)	5(3–7)	4(1–7)	3(1–6)	1(0–3)	4(2–6)	5(3–7)	27(18–38)
**England**	Back	Yes	45	8(6–9)	8(6–9)	6(3–8)	8(6–9)	7(3–8)	8(5–9)	7(5–9)	52(41–59)
		No	140	6(5–8)	6(3–8)	5(3–7)	5(2–6)	2(0–5)	5(2–7)	5(3–7)	52(41–59)
**Both countries**	All conditions	Yes	86	8(6–9)	8(6–9)	8(5–9)	8(6–9)	3(1–7)	8(5–9)	7(5–9)	51(39–58)
		No	424	6(4–7)	5(3–7)	4(2–7)	3(1–6)	1(0–3)	5(2–7)	5(3–7)	30(20–39)

† = not significant, all other scores are significantly different between SBT > 4 yes and no.

Table 3 show a significant difference in the median BQ sub-score between patients with SBT score ≥4 (Yes) and patients with SBT score <4 (No). Note the low score for anxiety and depression for the non-distressed by condition patients.

**Figure 2:**
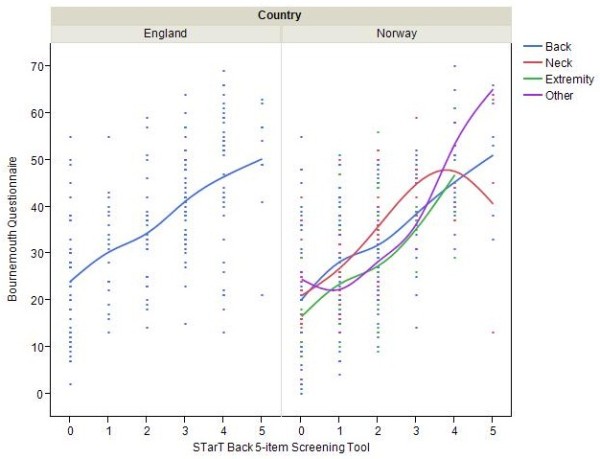
**Association between total BQ score and total SBT score.** The line of fit for the different areas of complaint is indicated in different colors.

There was a statistical association between the BQ total score and SBT total score for all areas of complaint in both study populations (Spearman’s rho; Norway: neck 0.66, extremity 0.56, other 0.7, back 0.54, UK: back 0.58. p < 0.05). All BQ- items were moderate or highly associated with SBT score as shown in Table 4 and Figure 2. Associations differed between areas of complaint and country (Table 4). The association was high between the depression sub-score in SBT and the low mood sub-score in BQ for neck pain (0.60), moderate for back pain (Norway 0.47, UK 0.32) and low for extremity (0.29). There was a high association for the pain sub-score in BQ and the bothersomeness sub-score in SBT for back pain Norway (0.56) and moderate for back pain UK (0.48) and neck pain (0.38) (Table 5). However, there was a low to moderate association between the anxiety BQ sub-score and the anxiety sub-scores from SBT that have been derived from the Hospital Anxiety and Depression Scale (SBT “not safe to be active” (back Norway 0.29, back UK 0.19) and SBT “worrying thoughts” (neck 0.48, extremity 0.26, back UK 0.45)). Results given for scores with p < 0.05.

**Table 4:** Correlation (Spearman’s rho) between SBT 5-item psychosocial subscale and BQ scores

**BQ items**	**Norway**				**England**
	**Neck**	**Extremity**	**Other**	**Back**	**Back**
Pain	0.39	0.41	0.36†	0.49	0.48
ADL physical	0.46	0.49	0.48†	0.38	**0.51**
ADL social	**0.52**	0.45	0.61†	0.35	**0.55**
Anxiety	**0.66**	0.46	**0.91**	0.43	0.47
Depression	**0.68**	**0.56**	**0.96**	0.41	0.4
Work	0.43	**0.58**	0.43†	0.36	0.44
Control	0.37	0.14†	0.55†	0.43	0.41
Total	**0.66**	**0.56**	**0.7**	**0.54**	**0.58**

* P < 0.05.

† = not significant.

*High associations with statistical significance are marked in bold.

Table 4 show the association between the different BQ items and total SBT score. Spearman’s rho >0.5 is classified as a high if ≥5, medium if 0.3-0.5 and low if <0.3.

**Table 5:** Correlation (Spearman’s rho) between the pain, anxiety and depression sub-items from BQ and SBT 5-item scale

	**SBT**	**Not safe to be active**	**Worrying thoughts (anxiety)**	**Pessimistic expectations**	**Not enjoy things (depression)**	**Bothersomeness**
**BQ**						
BQ1. pain	Neck	0.04	0.26*	0.31*	0.27*	0.41*
	Extremity	0.19	0.16	0.38*	0.1	0.38*
	Other	0.74*†	−0.22	0.28	0.21	0.31
	Back Norway	0.23*	0.17*	0.27*	0.29*	0.56*
	Back England	0.31*	0.25*	0.32*	0.32*	0.48*
	All conditions	0.21*	0.18*	0.31*	0.32*	0.48*
BQ4. anxiety	Neck	0.19	0.48*	0.31*	0.58*	0.51*
	Extremity	0.29	0.26*	0.36*	0.24	0.44*
	Other	0.31	**0.65**	0.83*†	0.73*†	**0.52**
	Back Norway	0.29*	0.13	0.41*	0.41*	0.38*
	Back England	0.19*	0.45*	0.28*	0.28*	0.35*
	All conditions	0.16*	0.33*	0.27*	0.40*	0.37*
BQ5. depression	Neck	0.26	0.58*	0.44*	0.60*	0.41*
	Extremity	0.46*	0.18	0.54*	0.29	0.29
	Other	**0.60**	**0.55**	0.81*†	0.76*†	**0.54**
	Back Norway	0.09	0.27*	0.26*	0.47*	0.30*
	Back England	0.09	0.33*	0.32*	0.32*	0.30*
	All conditions	0.13*	0.26*	0.38*	0.47*	0.30*

* P < 0.05.

*† Suspect p value due to small number (9) of the group “other”.

Table 5 show the association between the SBT items and the tree BQ items pain, anxiety and depression. The correlation coefficient is moderate to high between the SBT bothersomeness item and the tree BQ items. For most areas of complaint the correlation coefficient is moderate to high for BQ anxiety and depression vs SBT pessimistic expectations and depression. Spearman’s rho >0.5 is classified as a high if ≥5, medium if 0.3-0.5 and low if <0.3.

## Discussion

Our study has used SBT as a stratifying tool and showed that there is an association between high score on BQ and SBT - the higher the BQ, the higher the SBT score. We also see that patients who score higher than 40 on the BQ are most often also categorized as “distressed by condition” (>4) on SBT. Furthermore, those who score more than 4 on the depression item in BQ, also score high on the corresponding question on SBT in the “distressed by condition” group.

### Close association between BQ and SBT for depression scores

This study has provided a comparative analysis of the measuring properties of the SBT and the BQ for patients presenting to a chiropractic clinic. The BQ is only validated as an outcome measure. It is, as opposed to SBT, also validated to measure outcome of treatment for neck pain [11]. It has been developed to be a simple yet thorough tool to measure outcome of treatment. The psychosocial variables measuring anxiety and depression have shown poor responsiveness to change using BQ. Most likely because populations of patients presenting to chiropractic care have a low BQ baseline score (4 or below) for these items and consequently have too little change from baseline to follow-up when used as an outcome measure, i.e. a floor effect [16]. Here we have shown that these items may be of interest for the clinician if the patient present with a high score, especially for depression. This is apparent for all area of complaints included in this study. There is a low association between question 2 of the SBT and question 4 of the BQ addressing anxiety. The reason for this is unclear, but it is questionable if this SBT question actually represents the original anxiety component of the Hospital Anxiety and Depression Scale (HADS-Anx).

The study population are patients with a painful complaint and all groups had a fairly high pain score that was only slightly higher for patients with SBT score >4. There was a moderate to high association between the BQ pain question and the SBT bothersomeness question indicating that these items are related. Pain was less associated with the other SBT items and particularly not associated with the fear and anxiety items.

### Patient profile

A Danish study indicate a tenfold risk of psychosocial risk factors such as depression, catastrophizing and fear avoidance being present in the patients SBT identified as “high risk” [25]. Eleven percent was found to be in the high-risk group in this study of Danish LBP patients. This is comparable to what we found in our Norwegian population indicating a similar profile of chiropractic patients in Scandinavia. In contrast, the high risk group has been reported as high as 28% in an UK LBP population [5]. Our study shows a significant difference in the patient profile at baseline between chiropractic patients in Norway and England. Norwegian patients are somewhat younger, are less distressed by condition in general, have significantly lower catastrophization and depression scores, but are mildly more anxious than English patients. It therefore appears to be important that validation of diagnostic tools is restricted to the country it is being utilized within, as significant variations between countries are present.

In both countries there were more women than men seeking care. This concurs with findings from a Canadian chiropractic teaching clinic [26]. In the Canadian study they also reported that a majority of patients seeking care had spinal pain (81.4%) similar to the Norwegian population in our study where 66% presented with back pain and 21% with neck pain. One in five in the Canadian study had an extremity complaint and 10% cervical pain while in our study one in five had a cervical complaint and 10% extremity complaint. In a Belgian study of chiropractic clinics 91.5% of patients presented with spinal pain complaints [27]. In contrast, the prevalence of the different musculoskeletal areas of complaint to those presenting to general practitioners (UK) is quite different to that presenting to chiropractors, the most common area of complaint is low back (16%), and neck pain is fifth (9%) after knee, chest and shoulder complaint [28]. In a Norwegian epidemiological study [29], 34% of the population reported low back pain and 36% neck pain within the last week.

In our study we have shown that the neck pain, LBP and extremity pain patients score differently on BQ total, SBT total, “distressed by condition” and on the different sub-questions for both the BQ and the SBT. The group categorized as “other” in our study however, is small and therefore any statistical significance should be treated with caution. However, these patients show a tendency towards a more distressed psychosocial profile that should be explored in a larger study. There is a need to develop good stratification tools and outcome measures for generic use since as many as one in three seeking care has a complaint other than LBP as well as more than one area of complaint.

### Using questionnaires on different populations

The purpose of the SBT is to serve as a tool in deciding a treatment plan for patients presenting to primary care with low back pain, providing a more individualized treatment not based purely on the clinicians’ experience and expertise. Our study has found that the SBT psychological profile for LBP patients is similar to that found by other authors. The treatment package provided for the patient by different health care providers may differ in regards to addressing psychosocial issues depending on the clinician’s interest and formal training in treating musculoskeletal pain patients. This will influence the need to use stratification tools for referral but may give the qualified therapist a way to objectively target treatment for high risk patients.

As the BQ has never been used to stratify patients to “targeted treatment”, it is not possible to determine from our study whether the association between a high BQ score and high SBT score is clinically relevant. In a study assessing the SBT on chiropractic LBP patients showed that the high risk group had greater improvement and were equal to the median and low risk groups by the 30 day assessment [23]. Despite the high-risk group having greater pain and disability at baseline, they experienced greater improvement with regular chiropractic care. The chiropractic treatment paradigm may have aspects that address the psychosocial issues of the high-risk patient as measured by SBT, or the chiropractic patient population may differ from that of the general practitioners’. It is likely that the patients in both Field and Newell [23] and our studies were largely self-selecting and sought chiropractic care directly. In the UK population 41% of the patients had previously seen the chiropractor, and although this was not recorded for the Norwegian population or tested for, there is the possibility that an established therapeutic alliance may affect the patient’s psychological profile at different testing points.

The developers of SBT emphasize that an advantage of the tool is that it is validated for use in primary care, whilst other tools like the Oswestry Disability Index (ODI) and the Roland-Morris Disability Questionnaire are developed for use in secondary care. In the study by Hill and co-workers [5] the patients were referred to a physiotherapist, who then stratified the patient to “targeted treatment” through the use of SBT. Already at this stage in the stratification process the patient is once removed from the first point of contact in primary care. Evaluation of the implementation of diagnostic stratification tools in different areas of health care service may be of importance. This must be considered when using diagnostic and outcome instruments developed for use in a different health care settings than where it has been tested through RCTs.

Patients in primary care and secondary care may have different expectation to treatment success and thereby choice of treatment and this may also affect the baseline psychosocial profile of a patient population. In a study looking at minimal clinical important change in the ODI in a Danish population of LBP patients, one group treated by chiropractors in primary care and another group treated in the secondary sector of the Danish health care system, the investigators found a large difference in the reduction in ODI score needed for the patient to acknowledge a change. The percentage change scores from baseline were substantially higher in chiropractic patients in primary care (71%) compared to patients in secondary Care (27%) for a similar change in minimal important clinical change [30]. Although patients in primary care tend to be more acute and a rapid change in clinical presentation is expected, there may be an expectation of treatment outcome that are different for patients seeking chiropractic care.

BQ has never been validated as a stratifying tool, nor validated to identify predictors for chronicity. A study regarding BQ’s predictive and monitoring properties questioned its accuracy [12]. However, they did find that certain individual items were useful in predicting specific outcomes, making it still a useful predictive tool. They found that patients with a low score for BQ Work had a lower risk of being on sick-leave the following year. Patients scoring high on BQ Control had a higher risk of having LBP during the following year while a low score on this item or on BQ ADL predicted absence of debilitating LBP. They also found that a high score on BQ ADL, BQ Social and BQ Control predicted persistent LBP the following year. Although the use of BQ as a predictive instrument has been criticized [31] it is in use and has been found useful as part of a diagnostic package in clinical practice [32]. The generic use of BQ and SBT in general chiropractic practice is promising but should be used with caution until validated.

### Limitations

A validated Norwegian translation of the SBT was not available at the time of our study and may differ from the latest validated version. The SBT has received much attention and is of interest for health care decision makers. The informal translations available on the Keele University website are in clinical use. If the tool is applied on a population where it has not been properly validated it may have unforeseen consequences. We have here addressed the necessity of proper validation for both the generic versions of the SBT and the BQ. The public may assume that the translation process is of the same scientifically quality as the rest of the published work of the research group since the developers have approved translations through written permissions and posted them on their website.

The diagnostic grouping in the Norwegian population is crude, with the “other” group being small and heterogenic. There may also be a bias in which patient the participating chiropractor entered in the database as there was a large difference in number of patients provided from the Norwegian chiropractors. In future studies the distribution of diagnosis should be compared with the reimbursement registry for chiropractors to ensure that the data is representative for regular practice. In this study we did not analyze the influence of age and duration on the psychosocial profile in the different diagnostic groups.

For stratification tools and outcome tools to be of value in routine practice, they need to be easy to collect and evaluate for the clinician. The effort of manually collecting and logging the patient forms from paper may have been the reason why not all Norwegian clinics were able to submit data to the study, this in spite of the fact that it took less than one minute to enter a patient’ data set into the online survey database. Using an online data collection tool with automated data collection from the patient at baseline and follow up, such as the one used for data collection in the UK study population, could improve the utilization of questionnaires for clinical use as well as for research.

### Clinical relevance and further research

Clinicians using BQ routinely should be aware that patients with a high total score or with a high score on the depression question may have a psychosocial profile that should be taken into consideration when developing a treatment plan.

This study is the first to give a diagnostic and psychosocial profile of chiropractic patients in Norway. The results show that there is a significant difference in demographics and psychological profile between the Norwegian and UK chiropractic patients, showing that study results cannot necessarily be extrapolated between countries, prompting the need for research across borders, and caution when pooling data from different countries.

This study shows that both SBT and BQ generic versions for use in all musculoskeletal complaints may give valuable insight into the patient psychosocial profile and that there is a need for validation of the generic versions of these questionnaires.

## Competing interests

The authors declare that they have no competing interests.

## Authors’ contributions

All the authors participated in the planning of the study. OCK coordinated the data collection. LRL was responsible for the design in Norway and did the data analysis together with DN. JF was responsible for design and data-collection in UK. PMSI took part in the design in Norway and the translation process of BQ as well as drafted the manuscript. All authors have critically revised the manuscript and approved the final manuscript.
